# Lion and spotted hyena distributions within a buffer area of the Serengeti-Mara ecosystem

**DOI:** 10.1038/s41598-021-01518-6

**Published:** 2021-11-15

**Authors:** Stanslaus B. Mwampeta, Clay M. Wilton, Imani J. Mkasanga, Lusato M. Masinde, Peter S. Ranke, Eivin Røskaft, Robert Fyumagwa, Jerrold L. Belant

**Affiliations:** 1 Department of Biology, Norwegian University of Science and Technology (NTNU), Realfagbygget, 7491 Trondheim, Norway; 2 Global Wildlife Conservation Center, State University of New York College of Environmental Science and Forestry, 1 Forestry Drive, Syracuse, NY 13210 USA; 3 Michigan Natural Features Inventory, P. O. Box 13036, Lansing, MI 48933 USA; 4 Tanzania Wildlife Management Authority, P. O. Box 277, Bariadi, United Republic of Tanzania; 5 Centre for Biodiversity Dynamics, Department of Biology, Norwegian University of Science and Technology (NTNU), Realfagbygget, 7491 Trondheim, Norway; 6 Tanzania Wildlife Research Institute, P. O. Box 661, Arusha, United Republic of Tanzania

**Keywords:** Ecology, Conservation biology

## Abstract

Most large carnivore populations are declining due to anthropogenic activities including direct persecution, prey depletion, habitat loss and degradation. protected areas (PAs) can help maintain viable large carnivore populations; however, anthropogenic activities occurring near and within PA borders or edges can reduce their effectiveness. We investigated the influence of edge effects on abundance of lions (*Panthera leo*) and spotted hyenas (*Crocuta crocuta*) in Maswa Game Reserve (MGR), a part of the Serengeti ecosystem in northern Tanzania. We conducted repeated call-ins to attract and enumerate lions and hyenas at 20 stations in MGR during June–July 2017. We used *N*-mixture models to estimate hyena and lion abundance in relation to land cover and distance from the south-western MGR borders which are adjacent to villages. We found lowest lion and hyena abundances by the south-western border, with abundance of both species increasing toward the eastern border adjacent to Serengeti National Park. Lions were uniformly distributed among land covers whereas hyenas were more abundant in woodlands. We suggest that reduced lion and hyena abundance near human settlements was in response to depleted prey, due to human actions. We recommend ecologically compatible land uses and effective border patrols to mitigate these adverse effects.

## Introduction

Over 60% of large carnivore species are threatened with extinction and nearly 80% are experiencing population declines^1^, with remaining populations occupying reduced portions of their historic ranges. For example, lions (*Panthera leo*), are limited to 8% of their historic distributions^2^ and spotted hyena (*Crocuta crocuta*) populations, previously considered stable, are also declining^1^. Although little recognized, large carnivores provide important ecosystem functions, as their trophic position can influence ecological cascades and carbon sequestration^1,3,4^.Through interference interactions, large carnivores may regulate each other^5^, as well as prey and mesopredator populations^5^. Additionally, large carnivores may in part regulate diseases^6,7^ and provide scavenging opportunities for mesopredators and avian predators^8^.

As large carnivores are prone to conflict with humans^9^, viable populations often are maintained within protected areas (PAs)^10^. These PAs are increasingly effective when surrounded by land use compatible with wildlife^11,12^. Wildlife landscapes beyond PAs borders can serve as buffers and are critical to large carnivores due to their extensive space use and likelihood of ranging beyond core PAs^1^. Most PAs in Africa however, harbor large carnivore populations below their carrying capacity^13,14^. Several factors account for reduced populations including overexploitation (e.g., traditional medicines, illegal and poorly regulated hunting), depletion of prey species^9,14,15^, human encroachment resulting in habitat loss and degradation, and human-carnivore conflicts^14,16^. Overall, these and other human actions negatively influence not only large carnivore populations, but also the ecological integrity of PA and buffers^9^.

Human activities including illegal wildlife harvests, may adversely affect PAs and buffer areas^4,11,17,18^. For example, wildebeest (*Connochaetes taurinus*) biomass in Serengeti National Park (SNP) was reduced by at least 75% within 15 km of its border^4^. Wide-ranging movements of large carnivores result in their greater likelihood of crossing PAs borders, into unprotected zones with greater risk from human conflicts^9,19–21^.

Our objective was to assess whether lion and spotted hyena abundance varied as a function of distance from the border of Maswa Game Reserve (MGR). We conducted call-in surveys and used N-mixture models to estimate lion and spotted hyena abundance and distribution within MGR, a PA and buffer area within the Serengeti ecosystem of Tanzania. We predicted that lion and hyena abundance would increase as distance from PA edge increased, in particular on the south-western border of MGR where there is greater human habitation. Further, we expected this relationship to vary based on distribution of land covers, with greater hyena abundance in grasslands and lion abundance in forested areas^21–23^. Finally, we anticipated less tolerance by lions to human disturbances than by hyenas, as felids are typically more sensitive to anthropogenic disturbances than hyaenids and canids^17,24^.

## Results

**Figure 1:**
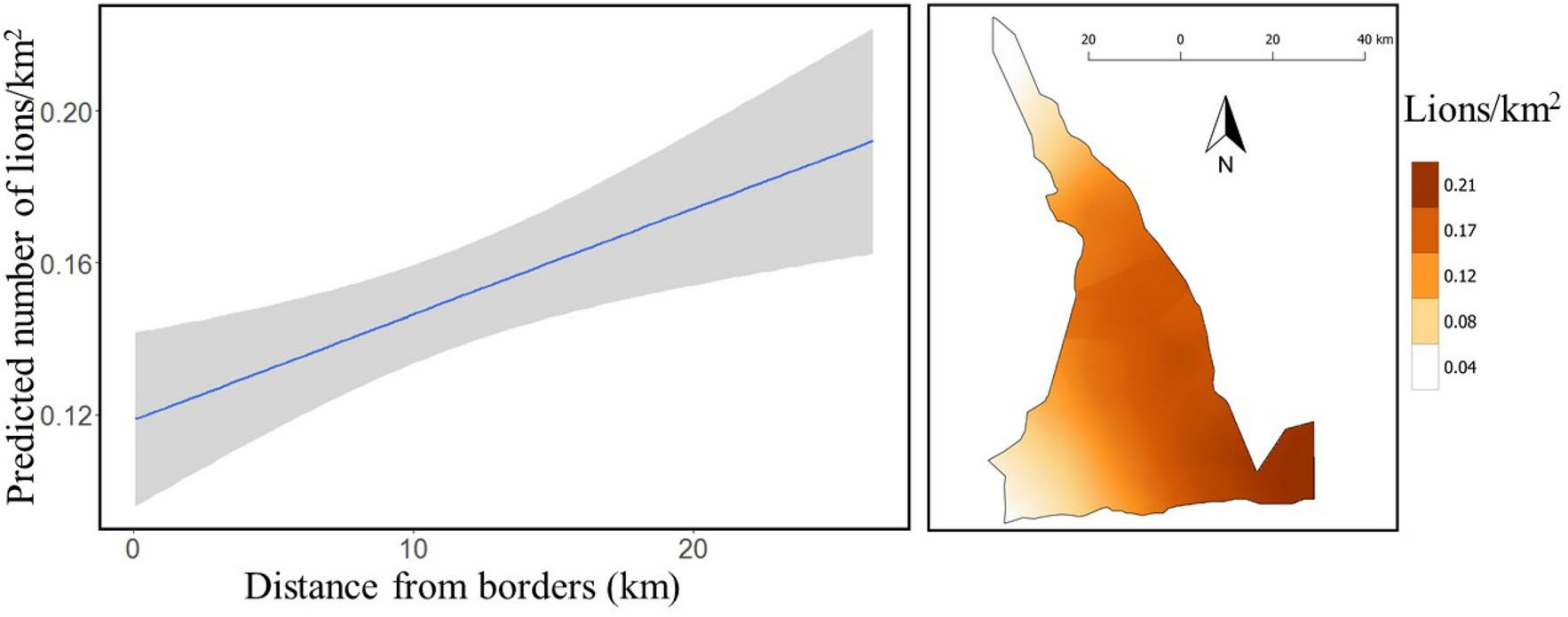
Estimated density of lions (left panel) in relation to distance from south and west borders and their spatial distribution (right panel), Maswa Game Reserve, Tanzania, 2017. Gray shading in left panel represents 95% credible interval.

We detected lions at 12 of 20 sites (60%) with maximum counts at these sites ranging from one (1) to 10 individuals. Model goodness-of-fit (GOF) was good, with a Bayesian p-value of 0.48. Detection probability of lions across sites varied from 0.10 to 0.50. Lion density was greater closer to SNP (mean = 0.146 lions/km^2^, SD = 0.062, 95% CI = 0.046–0.282) than near the south-western border of MGR (mean = 0.039 lions/km^2^, SD = 0.041, 95% CI = 0.001–0.148) (Fig. 1). Overall, we estimated 303 (SD = 40; 95% CI = 237–392) lions in MGR (0.138 lions/ km^2^). Land cover did not influence lion abundance and probability of detecting lions did not vary across weeks (Table 1).

**Table 1:** Covariates influencing abundance of lions and spotted hyenas, Maswa Game Reserve, Tanzania, June–July 2017.

Species	Covariate	Mean	SD	Rhat	Credible interval	
					2.5%	97.5%
Lion	**Distance to border (km)**	**0.005**	**0.003**	**1.000**	**0.000**	**0.010**
	Shrub	− 0.011	1.370	1.077	− 0.661	0.689
	Sparse grassland	− 1.123	1.123	1.000	− 3.766	1.661
	Dense grassland	0.155	0.448	1.009	− 0.702	1.056
	Woodland	0.285	0.337	1.010	− 0.351	0.952
	Closed grassland	0.170	4.112	1.008	− 7.440	8.683
	Shrub-grassland	− 0.031	0.478	1.099	− 0.938	0.927
Hyena	**Distance to border (km)**	**0.005**	**0.003**	**1.000**	**0.000**	**0.010**
	Shrub	− 0.165	0.595	1.001	− 1.329	1.016
	Sparse grassland	8.818	4.787	1.000	− 0.008	18.747
	Dense grassland	0.286	0.860	1.001	− 1.317	2.051
	**Woodland**	**1.632**	**0.411**	**1.002**	**0.833**	**2.447**
	Closed grassland	11.393	8.073	1.000	− 4.212	27.334
	Shrub-grassland	− 0.610	0.799	1.002	− 2.050	1.082

Significant covariates are indicated in bold font. Mean = estimate mean, SD = Standard Deviation, Rhat = Gelman–Rubin convergence diagnostic, convergence occurs at Rhat = 1.

**Figure 2:**
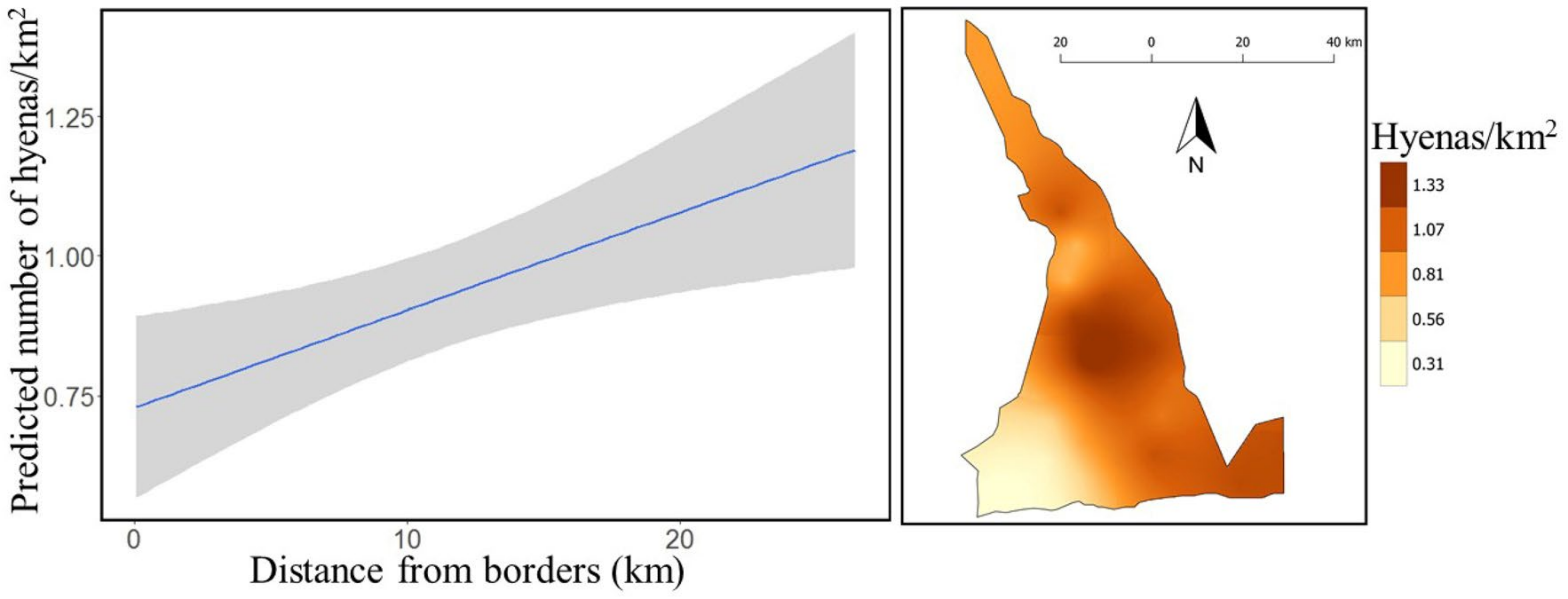
Estimated density of hyenas (left panel) in relation to distance from south and west borders and their spatial distribution (right panel), Maswa Game Reserve, Tanzania, 2017. Gray shading in left panel represents 95% credible interval.

We detected hyenas at all 20 sites (100%) with the maximum count ranging from one (1) to 27 individuals. Model GOF was good^25^, with a Bayesian p-value of 0.60. Hyena detection probability ranged from 0.47 to 0.66. Hyena abundance was greater further from the south and western borders (mean = 1.264 hyenas/ km^2^, SD = 0.183; 95% CI = 0.956–1.612) than at the border to human settlements; (mean = 0.300 hyenas/km^2^, SD = 0.167; 95% CI = 0.067–0.765) (Fig. 2). Overall, we estimated 2 008 (SD = 146; 95% CI = 1 747–2 319) hyenas in MGR (0.940 hyenas/ km^2^). We found significantly greater hyena abundance within woodlands and probability of detecting hyenas did not vary across weeks (Table 1).

## Discussion

We demonstrated that lion and hyena distributions varied across MGR with both species less abundant near the south-western border, increasing eastward toward the SNP border. Hyena abundance near the SNP border was more than four times higher (1.264 hyenas/km^2^) than at the south-western border (0.300 hyenas/km^2^). Although trophy hunting is allowed in MGR, it is unlikely that hunting caused the observed spatial variation in hyena and lion distributions. Four lions were legally harvested in MGR in 2013 and none since that year (MGR, unpublished data). Hyenas are undesirable as trophy animals and therefore rarely hunted in MGR (MGR, unpublished data).

Human disturbances are the probable cause of the observed gradual increase in numbers of lion and hyena from south-western to eastern borders of MGR. South-western areas of MGR receive limited patrols and therefore, daily incursions of poachers, agro-pastoralists, and livestock occur^4^. Veldhuis et al.^4^ reported high human encroachment in PAs within the Greater Serengeti Ecosystem (GSE) seven (7) km from the border. These areas within the GSE appear too degraded to support adequate prey biomass^4,26^ Depleted prey is likely due to poaching which is generally intense within the GSE; over 160,000 animals of various species are killed annually^10^, but poaching is greater in MGR because bushmeat from poaching is commercialized (i.e., 93% of poachers sell bushmeat^27^). Poaching and livestock incursions can also indirectly deplete the prey base of large carnivores^14,28,29^. Large carnivores are killed to reduce livestock depredations and in retaliation of livestock loss^4,11,30^. Finally, wire snares used for poaching are non-selective and can cause substantial carnivore mortalities (e.g., > 7.5% of adult hyena mortality in SNP^22^). Low prey density and poaching are therefore likely causes of lower hyena and lion abundance in areas of MGR near human settlements.

We provide the first abundance estimate for hyenas using *N*-mixture models which are robust as they can account for individual and group responses^31,32^. Our average density estimates of 0.940 hyenas/km^2^ is slightly greater than reported for SNP (0.600–0.800 hyenas/km^2^; Hofer and East, 1993^25^, 0.33–0.74 hyenas/km^2^; Durant et al*.*^33^). To our knowledge, there are no previous estimates for spotted hyenas in our study area. Less interference competition between lions and hyenas due to low lion abundance in MGR could explain greater hyena abundance in MGR than in SNP. Similarly, wild dogs (*Lycaon pictus*) have not reestablished in SNP, apparently in part due to interference competition with lions^34,35^. To our knowledge this is the first lion estimate for MGR. Our estimate of 0.138 lions/ km^2^ is slightly lower than estimates in grasslands of nearby southeastern SNP (0.144 lions/km^2^), using a similar technique^31^. Although woodland vegetation and year-around water availability would suggest greater lion abundance in this area^36^, human activities near the south-western borders of MGR appear to have reduced overall lion density in MGR. Because of low responses, our survey may have excluded most young individuals. But we believe that this has a minimal effect in potential management decisions as young animals are reproductively inactive, and especially for the lions, cubs are prone to higher mortality (up to 50%^37^).

Contrary to our second prediction we observed more hyenas in the woodlands of MGR, which differed from observed distributions in SNP and Maasai Mara National Reserve (MMNR), Kenya, where hyena distributions were greater in open habitats than woodlands^22,23,38^. The observed difference in hyena habitat use may be a consequence of adaptability and behavioral plasticity toward anthropogenic disturbances. Increasing human disturbances in MGR^4^ may have caused hyena to use woodlands and forests more often, which have fewer prey, but denser vegetation that may confer protection from humans^23,39^. This observation is congruent with our third prediction that hyenas are more adaptable to anthropogenic disturbances than lions. This finding is supported by previous works^40^, where livestock grazing in the core of hyena territories in MMNR caused hyenas to shift their activities to the peripheries of their territories^40^.

We found no evidence that land cover influenced lion distributions in MGR, similar to lions in SNP^31^. Although most carnivore distributions are positively associated with prey density and foraging opportunities^41^, lion distribution is influenced by prey availability and vulnerability^36,42^. We therefore suggest that lions are either more strongly influenced by other factors such as prey distribution or avoid encountering humans in more suitable habitats. Including more refined metrics of anthropogenic disturbances could further elucidate drivers of lion distributions.

Our study suggests that large carnivores are spatially depleted in south-western MGR. Similarly, Veldhuis et al.^4^ reported that wildebeest spend less time in these border areas. Only 11% of the historical Maswa region is currently protected^11,26^; poaching and overgrazing continue and are likely to further increase with increasing human population (4% annual population increase^43^). However, MGR acts as a migratory-wildebeest refuge during the short dry season (February–March) which extends through April in drought years^11^. As demonstrated in other buffer areas, MGR absorbs enormous anthropogenic pressure which could otherwise adversely affect more core PAs such as SNP^11,44^. Our findings agree with previous studies which suggest that Maswa is ecologically eroded^11^, which if not rapidly addressed may dramatically and further damage the integrity of the GSE.

Many large carnivore species are increasingly threatened^1,31^, and limitations to their effective conservation includes a paucity of information on population size and dynamics^2^. With a few notable examples, knowledge of Tanzania’s large carnivore populations, especially in hunted areas, is limited. Crosmary et al.^45^ estimated lion populations in Selous Game Reserve, but used a track-count method, which is widely criticized^31,46,47^. Call-in surveys are generally recommended^47–50^ and we further demonstrate that call-in surveys appear applicable for enumerating large carnivores in hunted populations, where poorly-regulated harvests may accelerate local declines^51^. When properly conducted, repeating estimates using our study design may influence management decisions, including setting appropriate harvest quotas^52^ and assessing the performance various harvest practices (e.g., age limit^53^).

A long-term solution is needed to address the loss of large carnivore habitat suitability in MGR. Human encroachment inside the reserve needs to be reduced and more compatible land-use practices should be encouraged among local people. These activities could include conservation compatible projects like bee keeping^10^. Effective patrols and additional ranger posts along the south-western border could reduce poaching and illegal livestock incursions. Importantly, management authorities in MGR can work toward further improving the efficiency of detections, reporting, and responding to illegal activities within the reserve.

## Material and methods

### Study area

**Figure 3:**
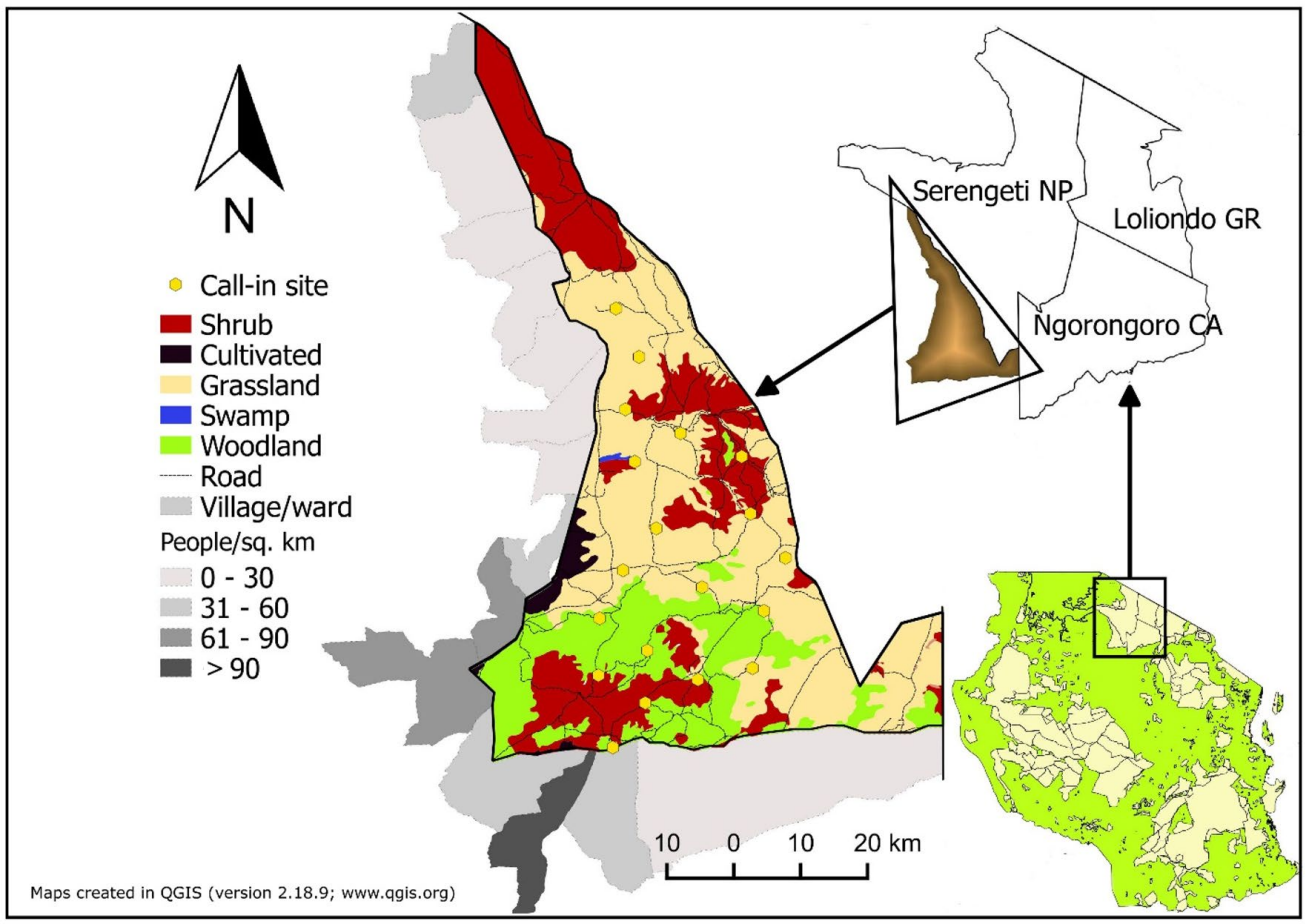
Locations of call-in sites used to estimate lion and spotted hyena abundance, Maswa Game Reserve, Tanzania, 2017. Beyond southern and western borders are the neighboring villages/wards. Light gray are less human populated villages/wards than darker grey.

We conducted this study in the dry season during June–July 2017 in the 2200 km^2^ MGR, Tanzania (Fig. 3). MGR is the south-western portion of the Greater Serengeti Ecosystem (GSE)^4^. GSE covers 25,000 km^2^ between Tanzania and Kenya. Comprising of SNP and MMNR as core areas, surrounded by buffers including MGR^4^. In the east, MGR borders and buffers SNP from several villages found on the western GSE^11^.Thirteen wards/villages borders MGR to the south and west, some of these villages are more densely populated than a national average (51 people/km^2^; Fig. 3^54^). Apart from SNP eastern MGR borders Makao Wildlife Management Area (WMA), and Ngorongoro Conservation Area (NCA). Annual rainfall in MGR increases from south (~ 550 mm) to north (~ 850 mm;^17^), with most precipitation occurring during November–May, with a short dry season in January–March^11^. Southern MGR is predominantly woodland whereas grasslands are interspersed with dense forest patches in the north^44,55^. MGR supports over two million migratory ungulates during February–March, but migration can occur through April during dry years^7,11^. Wildlife is subjected to legal harvests in MGR during July–December.

## Methods

We established 20 call-in sites throughout MGR, with sites separated by at least 8 km to ensure spatial independence and reduce double counting (Fig. 3;^34,48,50^). Because of limited nighttime accessibility, our call-in sites were on roads and we randomly selected the first site and systematically placed the remaining sites after each 8-km along the same or nearby road within MGR. We surveyed the 20 sites over five consecutive nights (4 sites each night) in 5 sessions for 5 consecutive weeks during June–July 2017. Typically, we broadcasted vocalizations during 1900–0300 h for 70-min per site following (Belant et al*.*^31,49^). We followed previously described procedures for broadcasting vocalizations [see Ref.^49^] using a lion roar, prey in distress, and spotted hyena whoop call. Prey in distress included warthog (*Phacochoerus africanus*) for sessions 1 and 4, wildebeest (*Connochaetes taurinus*) for sessions 2 and 5, and zebra (*Equus quagga*) for session 3 (see Belant et al.^49^). We broadcasted calls at up to 116 dB using a commercial game calling system (Foxpro Inc., Lewistown, Pennsylvania, USA) and 4 speakers mounted at 90-degree intervals on the roof of a vehicle about 2.4 m above ground.

We created a 3-km radius (28.27 km^2^) buffer around each call-in site and used GIS to estimate the percentage of land cover in each. We obtained GIS layers from the Serengeti-Mara database, managed by Tanzania National Parks and Frankfurt Zoological Society (https://serengetidata.weebly.com/). We categorized existing land covers into six classes including sparse grassland, closed grassland, dense grassland, shrub-grassland, shrubland, and woodland^31,56^ and incorporated these attributes in models as they are known to influence distributions of lions and hyenas^32,42^. We used a 3-km^2^ effective radius^50,57^ to convert abundance into density estimates.

We estimated hyena and lion abundance at call-in sites using *N*-mixture models^58–60^ in a Bayesian framework and compared estimated detection probabilities across sites. *N*-mixture models commonly assume closure in the studied population. We considered lion and hyena population size to be stable during the survey based on dry season stability of residence prey species^22^. The ‘true’ ecological state *N*_*i*_ describing abundance (i.e. number of individuals in the area of influence of our call-in sites) in site *i* was defined as a Poisson random variable, with an expected value *λ*_*i*_^58^. A site corresponded to the area of assumed influence of a call-in. We modeled the expected value of the Poisson distribution as a linear combination of an intercept *a*, and a random site effect *ε*_*i*_ on the log-scale as:

$$\begin{aligned} & N_{i} \sim Poisson\,\left( {\lambda_{i} } \right). \\ & {\text{Log}}\left( {\lambda_{i} } \right) \, = a \, + \varepsilon_{i} \\ \end{aligned}$$

Because lion and hyena responded to the call site as a group, individual detection was not completely independent. Therefore, we modeled the influence of this detection heterogeneity^25,54^, while accounting for imperfect detection. We modeled the count process *y*_*it*_ in site *i* during session *t* conditionally on the true abundance as:

$$y_{it} \sim beta{\text{-}} binomial\,(N_{i} , \, P_{it,} \rho ),$$

where *ρ* is a correlation parameter^44^, such that:

$$\rho = \frac{1}{\alpha + \beta + 1},$$

where *pit* is the individual detection probability in site *i* during week *t.* We allowed detection probability *pit* to vary among sites and sessions, following a non-informative uniform prior:

$$pit \sim\,{\text{Uniform}} \,(0, 1).$$

We next generated the mean session detection probability for 20 call-in sites. We then estimated the population size of each site by first accounting for potential sampling biases. *N*-mixture models typically rely on several assumptions including population closure, absence of false positives, and independence and homogeneity of detection^60^. Our sampling approaches mitigated any potential departures from these assumptions as we conducted this survey over a short duration (i.e., 5 weeks) with long distances (> 8 km) between call-in sites which reduced the potential of double counting. Further, we also noted the direction of individual approach and departure during call-ins, recording of individual age classes.

We developed Bayesian models for call-in counts using package “jagsUI”^61^ in program R version 4.0.3^62^, with non-informative priors for each parameter. We ran three chains of 100,000 iterations after a 10,000 burn-in with a thinning of 10 and monitored convergence of the MCMC chains using Gelman–Rubin convergence diagnostic (*R-hat,* at convergence *R-hat* = 1^63^). We assessed GOF of our model based on its derived Bayesian *p*-value (bpv), with values close to 0.5 suggesting good model fit^25^. We used a variable selection process for the regression model as our model selection criteria^64,65^. We present average estimated abundance at call-in sites, as well as corresponding detection probabilities with 95% credible intervals.

As the south-western boundary of MGR borders human settlements and agro-pastoralists. We used distance from call-in site to the nearest border (south or west), to model the influence of this edge effect on abundance of lions and hyenas. Human settlements and agro-pastoralism can influence animal distributions, including survival and increased conflicts with humans^3,9,11,18^. We simultaneously modeled potential effects of landcovers.

## Data Availability

Data available on the request from the authors.
